# A multicentre study investigating parameters which influence direct bacterial identification from urine

**DOI:** 10.1371/journal.pone.0207822

**Published:** 2018-12-11

**Authors:** Yuliya Zboromyrska, Jordi Bosch, Jesus Aramburu, Juan Cuadros, Carlos García-Riestra, Julia Guzmán-Puche, Carmen Liébana Martos, Elena Loza, María Muñoz-Algarra, Carlos Ruiz de Alegría, Victoria Sánchez-Hellín, Jordi Vila

**Affiliations:** 1 Consorci del Laboratori Intercomarcal, Vilafranca del Penedès, Spain; 2 Department of Clinical Microbiology, Hospital Clínic, School of Medicine, University of Barcelona, Barcelona, Spain; 3 ISGlobal, Instituto de Salud Global de Barcelona, Barcelona, Spain; 4 Microbiology Unit, Hospital Universitari Arnau de Vilanova, Lleida, Spain; 5 Microbiology Department, Hospital Príncipe de Asturias, Alcalá de Henares, Spain; 6 Microbiology Department, Complejo Hospitalario Universitario de Santiago de Compostela, Santiago de Compostela, Spain; 7 Microbiology Unit, Hospital Reina Sofía, IMIBIC-Reina Sofía University Hospital-University of Córdoba, Córdoba, Spain; 8 Infectious Diseases and Clinical Microbiology Unit, Complejo Hospitalario de Jaén, Jaén, Spain; 9 Department of Microbiology, Hospital Universitario Ramón y Cajal, Madrid, Spain; 10 Department of Clinical Microbiology, Hospital Universitario Puerta de Hierro, Majadahonda, Spain; 11 Microbiology Department, Hospital Universitario Marqués de Valdecilla, Santander, Spain; 12 Microbiology Department, Hospital General Universitario de Elche, Elche, Spain; Universitatsklinikum Hamburg-Eppendorf, GERMANY

## Abstract

Rapid diagnosis is one of the best ways to improve patient management and prognosis as well as to combat the development of bacterial resistance. The aim of this study was to study parameters that impact the achievement of reliable identification using a combination of flow cytometry and matrix-assisted laser desorption ionization time-of-flight mass spectrometry (MALDI-ToF-MS).The study was carried out in nine hospitals in Spain and included 1,050 urine samples with bacterial counts of 5x10^6^ bacteria/ml. MALDI-ToF-MS-based identification was performed according to a previously described protocol. Valid identification by direct MALDI-ToF-MS was obtained in 72.8% of samples, in 80.3% of samples found to be positive by culture, 32.2% of contaminated samples, and 19.7% of negative samples. Among the positives samples with a valid identification the concordance at the species level was 97.2%. The parameters related to success of direct identification were: high bacterial count, the presence of *Escherichia coli* as a pathogen and rod-bacteria morphology provided by flow cytometry. The parameters related to failure were a high epithelial cell (EC) count, a high white blood cell (WBC) count and urine samples obtained from in-patients. In summary, this multicentre study confirms previously published data on the usefulness and accuracy of direct MALDI-ToF-MS-based identification of bacteria from urine samples. It seems important to evaluate not only the bacterial count, but also other parameters, such as EC and WBC counts, bacterial species and morphology, and the health care setting, to decide whether the sample is suitable for direct identification.

## Introduction

Urinary tract infection (UTI) is one of the most frequent infections attended in primary care and hospital settings [1, 2]. UTI is an important cause of morbidity and mortality as well as one of the major causes of prescribing antimicrobial therapy[3, 4]. In recent years we have observed a rise in bacterial resistance, not only in hospitals but also in the community[5]. This makes the choice of appropriate empirical therapy difficult and could have a negative impact on patient outcomes. Therefore, there is a need for rapid diagnostic tools which could improve patient management and treatment, especially in patients with pyelonephritis and urinary sepsis, providing rapid and accurate information within a few hours.

Matrix-assisted laser desorption/ionization time-of-flight mass spectrometry (MALDI-ToF-MS) has become a standard method for bacterial identification (ID) in most of Clinical Microbiology laboratories, mainly in colonies of microorganisms grown on culture media. Although MALDI-ToF-MS can be used for ID from direct biological samples, it has several limitations. There is a need for sufficient numbers of bacterial cells and sample volume. The presence of human cells as well as proteins and other organic components requires sample preparation before analysis with MALDI-ToF-MS. Successful direct ID from urine samples has been previously reported[6, 7]. Nevertheless, urine is the most common sample processed in microbiological laboratories. The rate of negative samples can be of up to 60–70% of total urine samples received [8, 9]. Flow cytometry can classify and count different cells present in the sample, including human cells and bacteria. Currently this method is widely used for the detection of negative urine samples to avoid their cultivation [10, 11]. Several studies have shown the utility of flow cytometry to identify positive urine samples prior to MALDI-ToF-MS-based ID [12–14]. However, these were single-centre studies. In addition, parameters other than bacterial count provided by flow cytometry may be important and their impact on direct identification should be investigated. The aim of this multicentre study was to evaluate the efficacy and accuracy of a combination of flow cytometry and MALDI-ToF-MS for direct ID from urine samples, using a previously described protocol for direct ID [13] without changing the routine culture and identification procedure for each participant, and while also evaluating parameters that affect the achievement of direct identification for future improvements in the sample preparation protocol.

## Materials and methods

The study was conducted in two periods, from May to July and from September to November 2016 in nine hospitals in Spain (Hospital Universitari Arnau de Vilanova de Lleida (a median of 150 urine samples processed per day), Hospital General Universitario de Elche (80 samples per day), Hospital Universitario Marqués de Valdecilla (80 samples per day), Hospital Universitario Reina Sofía de Córdoba (120 samples per day), Complejo Hospitalario Universitario Santiago (250 samples per day), Hospital Universitario Principe de Asturias (190 samples per day), Hospital Universitario Puerta de Hierro de Majadahonda (200 samples per day), Hospital Universitario Ramón y Cajal (350 samples per day), Complejo Hospitalario de Jaén (100 samples per day). Six out of the nine participating centres used tubes with a preservative agent. Samples from all centres but one were transported with refrigeration. Patient data were collected and anonymized before the analysis was performed. Based on the 86.4% of reliable direct ID obtained in a previous study with samples with a bacterial count of 5,000 bacteria/μl by flow cytometry and considering a non-inferiority limit of 75% and admitting 10% of losses, it was estimated that 118 samples were needed from each participating centre [13]. Samples were collected, transported and processed for urine culture according to the routine protocol of each centre. During working hours, all samples received were processed by flow cytometry and samples that achieved the cut-off value of 5,000 bacteria/μl were randomly included in the study. Bacterial identification of colonies grown on culture media was performed using conventional techniques according to the routine procedure of each participating centre and included chromogenic media, the MicroScan system (Beckman Coulter, Spain), the Wider system (Francisco Soria Melguizo, Spain) and MALDI-ToF-MS, depending on the centre. Flow cytometry was performed using UF-1000i (Sysmex, Kobe, Japan) in accordance with the manufacturer’s instructions. All centres followed the guidelines of the Spanish Society of Infectious Diseases and Clinical Microbiology (SEIMC) for performing quantitative urine culture and criteria of positivity. Generally, a culture was considered to be positive when ≥ 100,000 colony-forming units per millilitre (CFU/ml) of one or two microorganisms were isolated, and negative, when no growth was detected. Among samples with < 100,000 CFU/ml, the number and species (commensal flora) of microorganisms isolated, as well as clinical data were taken into account to consider a culture as positive or contaminated. Samples with three or more microorganisms were considered as contaminated.

**Fig 1:**
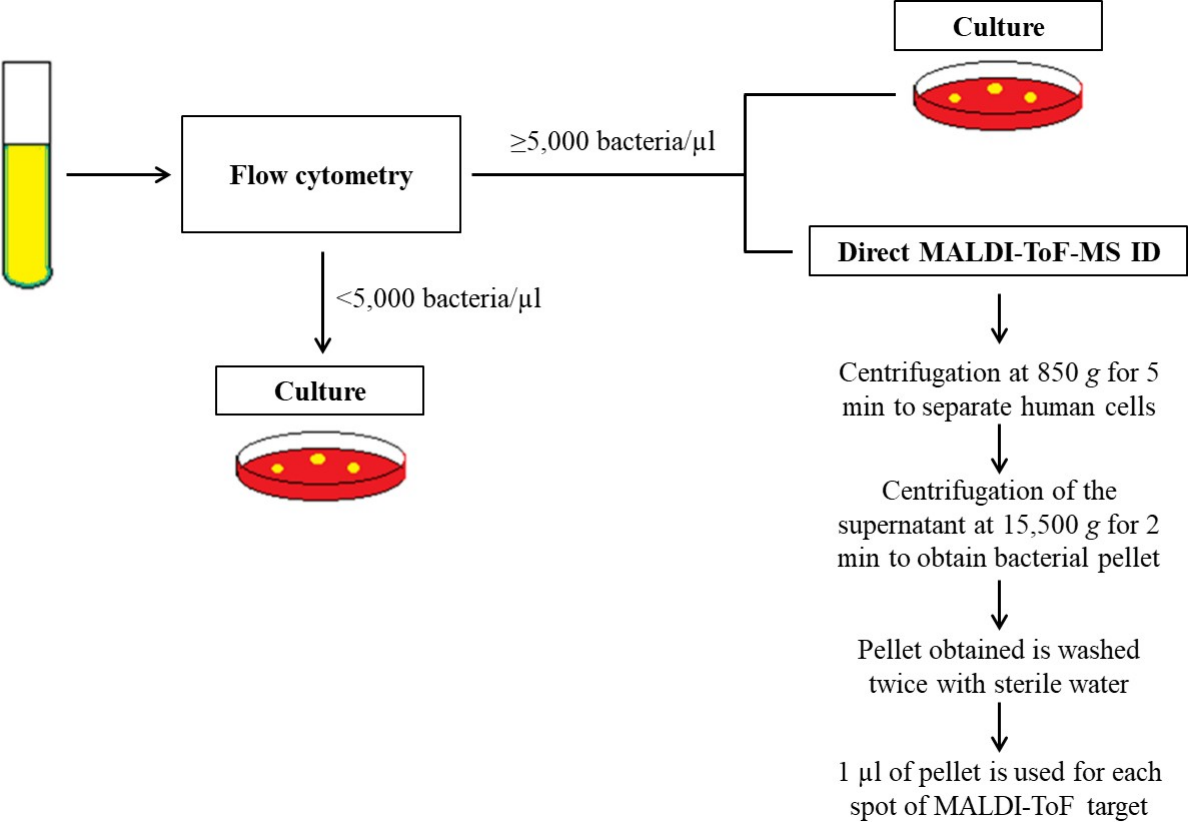
Scheme of the study protocol.

MALDI-ToF-MS-based identification was performed according to a previously described protocol [13]. Briefly, 10 ml of urine were centrifuged at 850 *g* for 5 min; then, the supernatant was centrifuged at 15,500 *g* for 2 min; the pellet obtained was washed twice with sterile water and used for ID by MALDI-ToF-MS (Fig 1). Each sample was processed in duplicate using two spots on a spectrometer plate. All but one of the centres used MALDI-TOF Microflex LT (Bruker DaltoniK GmbH). The remaining centre used VITEK-MS (bioMérieux, France). Identifications were carried out according to the manufacturer’s instructions. Valid ID for Microflex LT was considered if the same ID was obtained with a score of ≥ 1.7 for the first option from both spots or for the first two options from the list of one spot if the second spot did not provide a reliable identification or no peaks were detected. A valid ID for VITEK MS was considered when the two spots provided the same species identification with a confidence value of 99.9%.

### Statistical analysis

Descriptive statistics, including mean, standard deviation (SD), median and range for continuous variables and frequency and percent for categorical variables are provided by group. Statistical significance (*P*-value) between two independent subgroups was obtained using the Fisher's exact test for categorical variables and the Student's t-test for continuous variables. If the analysis of variance assumptions were violated (e.g. non-normality or heterogeneity) the comparison of continuous variables was performed using non-parametric tests: the Mann–Whitney U test in the case of two subgroups or the Kruskal-Wallis test in the case of more than two subgroups. A *P* value less than 0.05 was considered statistically significant. Multivariate logistic regression was applied by stepwise selection and adjusting for the effect of the centre to obtain the parameters associated with valid direct ID in positive urine samples. The effect of each parameter is presented with an odds ratio and 95% confidence interval.

### Ethical approval

The study was reviewed and approved by the Hospital Universitari Arnau de Vilanova de Lleida Institutional Review Board (IRB), acting as the single reference IRB (29/06/2016). The study was carried out without additional intervention of patients. All the samples were processed for the diagnosis of UTI as ordered by clinicians, and patient data were anonymized before the analysis. Informed Consent was not required.

## Results

After excluding samples with lost data, a total of 1050 urine samples were analysed. Table 1 shows the main characteristics of the samples included. A total of 224 (23.2%) samples were from male and 806 (76.8%) from female patients. Of 902 samples positive by urine culture, 870 (96.5%) had ≥10^5^ CFU/ml, 30 (3.3%) samples had ≥10^4^ CFU/ml and two samples (0.2%) had ≥10^3^ CFU/ml.

**Table 1:** Characteristics of patients and samples included in the study.

	Total	Male	Female	*P*-value
Age				
Mean (SD)	62.8 (21.7)	67.6 (17.2)	61.3 (22.7)	<0.0001^b^
95% CI	(61.5; 64.1)	(65.4; 69.7)	(59.8; 62.9)	
In/out-patients				
In-patients	192 (18.3%)	72 (29.5%)	120 (14.9%)	<0.0001^a^
Out-patients	858 (81.7%)	172 (70.5%)	686 (85.1%)	
Sample collection*				
Midstream	980 (94.1%)	205 (85.8%)	775 (96.6%)	<0.0001^a^
Indwelling catheter	35 (3.4%)	21 (8.8%)	14 (1.8%)	
Catheterization	13 (1.2%)	6 (2.5%)	7 (0.9%)	
Urostomy	13 (1.2%)	7 (2.9%)	6 (0.7%)	
Urine collection system with a preservative				
No	428 (40.8%)	112 (45.9%)	316 (39.2%)	0.0363^a^
Yes	622 (59.2%)	132 (54.1%)	490 (60.8%)	
Urinary tract infection (UTI)**				
Uncomplicated UTI	616 (79%)	111 (68.5%)	505 (81.7%)	NA
Pyelonephritis	73 (9.4%)	27 (16.7%)	46 (7.4%)	
Prostatitis	10 (1.3%)	10 (6.2%)	-	
No UTI	81 (10.4%)	14 (8.6%)	67 (10.8%)	
Urine culture				
Positive	902 (85.9%)	213 (87.3%)	689 (85.5%)	0.0462^a^
Contaminated	87 (8.3%)	24 (9.8%)	63 (7.8%)	
Negative	61 (5.8%)	7 (2.9%)	54 (6.7%)	
Mono/polymicrobial urine culture				
Total of positive urine cultures	902	213	689	
Monomicrobial	857 (95%)	197 (92.5%)	660 (95.8%)	0.0699^a^
Polymicrobial	45 (5%)	16 (7.5%)	29 (4.2%)	
Cell count by flow cytometry				
Epithelial cells				
Median (Range) cells/μl	6.5 (0–495.6)	3.6 (0–447.1)	7.9 (0–495.6)	<0.0001^c^
Red blood cells				
Median (Range) cells/μl	25.8 (0–4706)	39 (0–4706)	22.7 (0–2612)	<0.0001^c^
White blood cells				
Median (Range) cells/μl	194 (0–48,887)	552 (1–33,128)	146 (0–48,887)	<0.0001^c^
Bacteria				
Median (Range) bacteria/μl	20,206 (5002–454,734,000)	18,703 (5029/12,000,000)	20,518 (5002/454,734,000)	0.3513^c^

*Data available for 1041 samples: 239 from males and 802 from females

**Data available for 780 samples: 162 from males and 618 from females

^a^ Fisher's exact test

^b^ Student's t-test

^c_^Mann–Whitney U test.

SD: standard deviation; CI: confidence interval

Pathogens isolated from positive urine samples were: *Escherichia coli* (70.6%), *Klebsiella* spp. (16%), *Proteus* group (including species of *Proteus*, *Morganella* and *Providencia*) (4.7%), *Enterococcus* spp. (2.7%), *Enterobacter* spp. (2%), *Pseudomonas* spp. (1.1%) and others (2.9%). There were significant differences in the percentage of uropathogens between males and females for *E*. *coli* (61% vs. 73.6%, *P* = 0.0006) and *Enterococcus* spp. (5.2% vs. 1.9%, *P* = 0.0143).

On comparing in-patients and out-patients, there were no significant differences in the rates of positive, negative and contaminated samples. However, samples with the isolation of two pathogens were more frequent among in-patients (8.6% vs. 4.1%, *P* = 0.0196). Regarding the aetiology, microorganisms from the *Proteus* group and enterococci were more frequent among in-patients (8% vs. 3.8%, *P* = 0.0261, and 5.2% vs. 2.1%, *P* = 0.0328, respectively) whereas *E*. *coli* was more frequent among out-patients (62.6% vs. 72.5%, *P* = 0.0123). The rate of reliable direct ID was 73% among in-patients vs. 82% among out-patients. Valid ID was obtained in 84.3% of patients with positive urine culture and recorded a diagnosis of pyelonephritis (59 out of 70 with positive culture).

Table 2 shows the differences in cell count among positive, negative and contaminated samples.

**Table 2:** Cell count (median (range)) according to the positivity of the urine sample.

Median (Range) cells/μl	Positive (902)	Contaminated (87)	Negative (67)	*P*-value
**EC**	5.7 (0–447.1)	20.2 (0.2–495.6)	33.4 (0.5–167.7)	<0.0001^a^
**RBC**	24.5 (0–4,706)	48.2 (2.1–1525.6)	27.9 (2.5–421.6)	0.0005^a^
**WBC**	194 (0–48,887)	253 (3–18,505)	168 (4–13,046)	0.4313^a^
**Bacteria**	21,752 (5,002–454,734,000)	12,401 (5,124/84,439)	8,016 (5,010/91,119)	<0.0001^a^

^a^ Kruskal-Wallis test

EC: epithelial cells; RBC: red blood cells; WBC: white blood cells

Valid ID by direct MALDI-ToF-MS was obtained in 72.8% of samples, in 80.3% of samples positive by culture, 32.2% of contaminated samples, and 19.7% of negative samples. Among positive samples with valid ID the concordance at the species level was 97.2% and 98.3% at the genus level, and 12 samples (1.7%) showed a discordant result with the conventional ID (Table 3).

**Table 3:** Results of routine identification and MALDI-ToF-MS direct ID in 902 culture positive samples.

Species	Positive samples with valid direct ID (n = 724)	Concordant samples between routine and direct ID (n = 712)	Species level direct ID among concordant samples (%)	Positive samples with no valid direct ID (n = 178)
*Escherichia coli*	530	524^a^	524/524 (100)	107
*Klebsiella* spp.	117	114^b^	111/114 (97.4)	27
*Proteus* group	31	31	31/31 (100)	11
*Enterobacter* spp.	15	14^c^	12/14 (85.7)	3
*Enterococcus* spp.	10	8^d^	8/8 (100)	14
*Citrobacter* spp.	6	6	6/6 (100)	2
*Pseudomonas* spp.	6	6	5/6 (83.3)	4
*Serratia marcescens*	3	3	3/3 (100)	0
*Streptococcus* spp.	2	2	2/2 (100)	3
*Aerococcus* sp.	1	1	0/1 (0)	2
*Staphylococcus* sp.	1	1	1/1 (100)	4
*Raoultella ornithinolytica*	1	1	1/1 (100)	0
*Acinetobacter baumannii*	1	1	0/1 (0)	0
*Oligella urethralis*	0	-	-	1

^a^Discordant direct ID for 6 *E*. *coli* included 2 *Lactobacillus* spp., 1 *Bifidobacterium breve*, 1 *Gardnerella vaginalis*, 1 *Aerococcus* sp. and 1 *Klebsiella pneumoniae*

^b^Discordant direct ID for 3 *Klebsiella* spp. included 1 *Raoultella ornithinolytica*, 1 *Pseudomonas* sp. and 1 *Aerococcus* sp.

^c^Discordant direct ID for 1 *Enterobacter aerogenes* was *Klebsiella* pneumoniae

^d^Discordant direct ID for 2 *Enterococcus faecalis* included 1 *Raoultella ornithinolytica* and 1 *Lactobacillus* sp.

Twelve samples (19.7%) with valid ID and negative by culture included: 4 *E*. *coli*, 1 *Aerococcus urinae*, 1 *Actinotignum schaalii*, 1 *Gardnerella vaginalis*, 1 *Streptococcus* sp., 1 *Lactobacillus* sp., 1 *Alloscardovia omnicolens*, 1 *Corynebacterium riegelii* and 1 *Veillonella ratti*.

Interestingly, on analysing a total of 1050 samples, we obtained a higher rate of valid direct ID among samples without than with a preservative (76.4% vs. 70.4%, *P* = 0.0342). Nevertheless, regarding only positive samples by culture, there was no significant difference between the two groups (81.2% vs. 79.6%, *P* = 0.5562).

Table 4 shows the parameters associated with valid direct MALDI-ToF-MS ID on multivariate analysis.

**Table 4:** Parameters associated with valid direct identification in positive urine samples.

	OR	CI 95%	*P*-value
Centre			<0.0001^a^
In-patients	0.516	0.314–0.848	0.0090
*E*. *coli*	2.073	1.386–3.099	0.0004
Bacterial morphology as Rods	2.292	1.299–4.045	0.0042
Log EC	0.849	0.737–0.977	0.0227
Log WBC	0.701	0.628–0.782	<0.0001
Log bacteria	2.206	1.662–2.928	<0.0001

^a^this variable was included in the analysis as the percentage of valid ID was different among centres

OR, Odds ratio; CI, Confidence Interval; EC: epithelial cells; WBC: white blood cells

Additionally, we analysed the accuracy of bacterial morphology provided by flow cytometry in 835 of 902 positive samples (92.6%), with 83 samples being reported as cocci/mixed and 752 as rods. In 67 positive samples no bacterial morphology was provided. The global concordance was 92.1%. The sensitivity, specificity, positive predictive value and negative predictive value was 92.8%, 73.3%, 98.9% and 27.5%, respectively for cultures positive for bacilli or mixed cultures that included bacilli (Table 5).

**Table 5:** Concordance between bacterial morphology provided by flow cytometry and culture results.

Number of samples according to morphology	Rods by flow cytometry (n = 752)	Cocci/mixed by flow cytometry (n = 83)
Bacilli on culture (n = 786)	728	58
Bacilli and cocci mixed culture (n = 19)	16	3
Cocci on culture (n = 30)	8	22

## Discussion

The usefulness of a combination of flow cytometry and MALDI-ToF-MS for direct identification of pathogens in urine samples has been reported previously. The percentage of accuracy in bacterial identification directly from the sample depends on several factors, with bacterial count being the main factor. Samples with positive urine culture but with <10^5^ CFU/ml show a lower percentage of reliable direct identification compared to those with ≥10^5^ CFU/ml [7, 15, 16]. Another variable that negatively influences direct identification is the presence of a second pathogen. Wang *et al*. showed that the ratio of bacterial count between two pathogens determines the possibility of a reliable identification of one of the pathogens [15]. This could explain cases of valid identification obtained in contaminated samples due to the presence of a dominant microorganism. From the multivariate analysis, we showed that the presence of a high count of human cells such as WBC and EC associated negatively associated with a reliable identification. Our data suggest that more sophisticated protocols of sample preparation should be used if a high WBC count is detected by flow cytometry. Sanchez-Juanes *et al*. proposed the treatment of urine samples with SDS solution to enhance direct identification [17]. Previous studies also demonstrated better results, if protein extraction was applied to the bacterial pellet [7, 18]. Veron *et al*. compared a filtration-based sample preparation protocol with a differential centrifugation method and 5 hours of bacterial cultivation, obtaining better results with the filtration protocol [19]. Recently, Kitagawa *et al*. implemented ultrasonic baths to disperse bacterial aggregation prior to the centrifugation steps, improving the final identification [20]. Another approach for the samples with a high bacterial as well as WBC count could be a few hours of subculture on a solid medium prior to MALDI-ToF-MS ID [21]. The presence of samples with a high EC count could indicate poor sample collection quality, and these samples should not be processed for direct ID due to the increased probability of contamination.

In the present study we used a lower score than recommended by the manufacturer to consider a species ID as being reliable with MALDI-ToF-MS (1.7 vs 2.0). Although this could be criticized, according to the experience of the authors, species ID with scores ≥ 1.7 usually provide a reliable species level ID. Taking into account that we work with direct biological samples with a complex composition, lower scores can be expected compared with colony-based ID. In the study published by March Rosselló *et al*., the authors demonstrated that a lower score of even less than 1.7 could be considered for direct ID from urine samples [18].

While samples from in-patients showed a lower probability of valid direct ID, the rate of valid ID was sufficiently high to justify its application. Moreover, in this population, rapid ID may have a greater impact on patient management, as the percentage of resistant microorganisms, as well as species other than *E*. *coli* is higher [22].

We also analysed the presence of preservative substance in sample collection tubes. Preservatives help to avoid cell destruction and bacterial proliferation [23, 24]. In the absence of preservatives, contaminating flora may grow, while human cells are destroyed. This could explain the higher percentage of valid ID in samples without a preservative, when we analysed a total of samples, including contaminated ones. Among positive samples there were no significant differences in the percentage of valid ID between these two groups. Therefore, the use of a preservative could avoid valid direct ID of contaminated samples without affecting the rate of positive samples identified. Therefore, we recommend the use of preservatives for samples that cannot be processed immediately (mainly samples from primary care), especially if an efficient cold chain during transport is not assured.

Of 12 samples with discordant ID, in seven an identified microorganism usually grows with difficulty in conventional media. These directly identified microorganisms were probably present in the urine specimen in a higher concentration than the pathogen detected by the conventional method. Five of the remaining discordant results were probably due to erroneous ID by direct MALDI-ToF-MS or the routine method. Unfortunately, these discordant results were not confirmed by 16S rRNA gene sequencing, as has been done in previous studies [15, 25]. Regarding negative samples with valid ID, four *E*. *coli* cases could be explained by the therapy received (data not collected in this study). Other microorganisms showed fastidious growth, and most seemed to be contaminant microorganisms.

Regarding the data on bacterial morphology, we obtained an acceptable positive predictive value for bacilli. Nevertheless, we included only samples with a high bacterial count, and the number of samples which were positive for cocci was low in this study. Previous data support better results in predicting bacillary morphology with flow cytometry [26, 27].

Although the combination of flow cytometry and MALDI-ToF-MS could improve patient management, especially in a hospital setting, direct identification includes many manual steps that may impair implementation of this approach in routine laboratory work. Flow cytometry analysis takes about one minute, but the median time required to prepare one sample for MALDI-ToF-MS is about 20 min. Until a more automated process of sample preparation is established, direct identification should only be used in some cases with great clinical impact, such as those suspected of urinary sepsis and pyelonephritis.

Our study also suggests that different approaches should be studied and used for sample processing depending on the presence of EC or WBC. Since a high EC count may indicate probable sample contamination during sample collection, direct ID is not recommended due to the risk of obtaining false positive results by contaminating flora. A high WBC count is a good predictor of infection but may impair direct ID. We likely need to perform additional studies to determine the WBC count cut-off in order to apply more rigorous sample preparation protocols including the use of agents such as SDS to enhance cell lysis or even opting for short subcultures prior to MALDI-ToF-MS.

In summary, the results of this multicentre study confirm previously published data about the usefulness and accuracy of direct MALDI-ToF-MS ID from urine samples. It was of note that we observed an excellent concordance between direct and routine ID in all the participating centres without changing local urine processing protocols. Analysis of all the parameters provided by flow cytometry is useful for evaluating the quality of sample collection, the feasibility of direct MALDI-ToF-MS ID and for deciding upon the most adequate sample preparation protocol to be applied. Direct ID also allows the application of methods for antimicrobial susceptibility testing or resistance detection from a direct clinical sample once a reliable ID is obtained [13, 14].

## Supporting information

S1 Appendix Original database. (XLSX)
